# A Green Route for Substrate-Independent Oil-Repellent Coatings

**DOI:** 10.1038/srep38016

**Published:** 2016-11-29

**Authors:** Li-Ping Xu, Da Han, Xiuwen Wu, Qingqing Zhang, Xueji Zhang, Shutao Wang

**Affiliations:** 1 Research Center for Bioengineering and Sensing Technology, School of Chemistry and Biological Engineering, University of Science & Technology Beijing, Beijing 100083, P.R. China; 2 Key Laboratory of Bio-inspired Materials and Interface Science, CAS Center for Excellence in Nanoscience, Technical Institute of Physics and Chemistry, Chinese Academy of Sciences, Beijing 100190, P. R. China

## Abstract

Oil repellent surface have lots of practical applications in many fields. Current oil repellent coating may suffer from limited liquid repellency to oils or environmental risks. In this work, we report an eco-friendly ‘green’ processes for preparing oil-repellent surface using a renewable and environmentally benign bioresource alginate. The oil-repellent coating was prepared by a two-step surface coating technique and showed stable oil repellency to many kinds of oils. The fabrication process was very simple with no need for special equipment, and this approach can be successfully employed to various substrates with different compositions, sizes and shapes, or even substrate-independent oil-repellent materials. The as-prepared coating of calcium alginate may have a good future in packaging oil-containing products and foods.

Unwanted oil sticking is currently a limiting factor in many fields from industry to kitchen^1^,^2^,^3^. Oil-repellent surfaces are highly desirable and have potential applications in marine antifouling coating, oil/water separation, industrial metal cleaning, bioadhesion, microfluidic technology and food containers^4^,^5^,^6^,^7^,^8^,^9^,^10^,^11^,^12^,^13^,^14^,^15^,^16^.

Although intense research has been made in the development of liquid repellent surfaces^17^,^18^, existing surfaces show limited repellency to oils. Recently, several groups designed slippery superoleophobic surfaces by using porous fluorinated membrane to lock in place the infused fluorinated fluid^3^,^5^,^19^,^20^,^21^,^22^,^23^,^24^,^25^,^26^. However, they are always associated with biological and environmental risks owing to their toxicity and non-biocompatibility of fluorochemicals^27^. An ideal oil-repellent coating should have stable oil repellency to many kinds of oils, should be able to be deposited in a simple process on materials regardless of their size, shape, or composition. And the chemicals used in the fabrication of oil-repellent coating should be environment-friendly. The oil-repellent surfaces inspired by the underwater superoleophobicity of fish scales shows satisfied repellency to oils, which may bring new insights in fabricating novel oil-repellent coatings^28^,^29^,^30^,^31^,^32^,^33^.

Alginate is a naturally occurring poly-anionic polysaccharide derived from brown marine algae and recognized as safe substance (Food and Drug Administration)^34^,^35^ and having numerous applications in the biotechnology industry as non-toxic food additive, thickening agent, gelling agent, emulsifier and colloidal stabilizer^36^. Alginate can crosslink with polyvalent metal cations like Ca^2+^ to produce strong gels or insoluble polymers^37^,^38^. It is a renewable and environmentally benign bioresource. Moreover, its low-cost, nontoxicity, good biocompatibility and biodegrability made it ideal for “Green Chemistry” approaches. In this work, we report an eco-friendly ‘green’ processes for preparing oil-repellent surface using this natural and biodegradable polymer. No special equipment is necessary for this method, and the material components are readily available. Additionally, this approach could be successfully employed to various substrates with different compositions, sizes and shapes, or even substrate-independent oil-repellent materials. The as-prepared coating of calcium alginate may have a good future in packaging oil-containing products and foods.

The calcium alginate coatings were prepared according to the following steps. The solution of CaCl_2_ was casted onto the substrates and then dipped into sodium alginate solution for 10 min until the gel formed. In the process of gelation, CaCl_2_ is a cross-linker, and it is hypothesized that the CaCl_2_ can also be trapped in the substrate, leading to the formation of a stronger hydrogel layer at the surface, and CaCl_2_ anchors hydrogel to the surface even in underwater environment^39^. The morphology of calcium alginate coatings were characterized using scanning electron microscopy (SEM). SEM image (Fig. 1a) demonstrates that calcium alginate coating is composed of nanofibers, and those nanofibers randomly interconnect to form nanopores. The wetting properties of water and oil on calcium alginate coating were characterized comprehensively. Water contact angle (WCA) measurement indicated that the calcium alginate coating exhibits superhydrophilicity with WCA of 0° (Fig. 1b). Oil wettability of the obtained calcium alginate coating was evaluated by using an oil droplet (1,2-dichloroethane, DCL) as a detecting probe. As shown in Fig. 1c, the oil contact angle (OCA) is about 33.7 ± 6.2°. The calcium alginate coating can’t repel the DCL oil droplet due to the low surface tension of oil droplet. Then underwater OCA of calcium alginate coating was also measured. The DCL droplet was supported on the substrate as ball without spreading out as shown in Fig. 1d, and the measured underwater OCA is about 159.3 ± 2.2°. When the calcium alginate coating was immersed in the water, water can be trapped in those superhydrophilic nanopores. The trapped water acts as a repulsive cushion to the oil and thus an oil/water/solid three-phase system with Cassie state was formed. As a result, the calcium alginate coating exhibits underwater superoleophobicity in three-phase system by introducing a repulsive liquid (i.e. water in this case) into the porous surface. Figure 1e indicates that the calcium alginate coating presents unique underwater superoleophobic characteristics for various oils and organic solvents, including 1, 2-dichloroethane, silicone oil, thrichloromethane, edible oil and N-decyl hydride. All the underwater OCAs on the calcium alginate coating are larger than 150°, confirming the underwater superoleophobic properties.

The sliding property of oil droplets on calcium alginate modified glass (CA-glass) was evaluated underwater. The DCL oil droplet was used as the model oil. As shown in Fig. 2a, when a DCL oil droplet was brought into contact with the substrate with sliding angle of less than 2°, the oil droplet slid across and off the substrate immediately within a few seconds. The absorbing and holding of water in calcium alginate coating provide a liquid interface and is critical for the easy sliding of oil droplet.

This easy-sliding property of the calcium alginate modified surfaces can provide superior self-cleaning because the sliding water droplets are likely to take away dust deposited on the surface. The self-cleaning property was studied by choosing silicone oil as the dirt. Silicone oil droplet could easily be removed by water rinsing from water-swollen CA-glass in several seconds, while sticking of oil on bare glass was observed (Fig. 2b). The weak interactions among the oil droplet and the water-swollen CA-glass coating determine the self-cleaning property.

Different materials possess different surface properties and it remains an issue to develop a versatile approach that can fabricate oil-repellent coatings onto various material surfaces. To verify the versatility of this method, several substrates from steel foil, silicon wafer, glass slide, polyethylene terephthalate (PET) film to mica film (Fig. 3) were employed. Due to its high transparency, the calcium alginate coating brought little change to the appearance of various substrates. The oil wettability of coated substrates were assessed. With calcium alginate coating, all substrates exhibit stable underwater superoleophobicity with underwater OCA larger than 150°. The underwater OCA values (Fig. 3) changed after the coating in all cases, demonstrating that calcium alginate films can be formed on a wide variety of substrates. A further advantage of calcium alginate coating is that the coating is not limited by the size and shape. As shown in Fig. S1, highly curved surface such as glass beads or glass rods were coated with calcium alginate coating. Not easily accessible objects such as the inner side of bottles are hard to coat, and most commonly used coating methods including spin-coating, spraying, casting are not feasible. By introducing CaCl_2_ and sodium alginate solution into those bottles successively, the inner side of bottle could also be coated with calcium alginate coatings successfully.

Besides its versatility in coating different substrates, free-standing CA film can also be prepared. In last decades, the fabricating of substrate-independent oil-repellent materials is still a challenge^40^,^41^ although many self-cleaning and oil-repellent surfaces have been fabricated. Those films are adherent to the substrate surface and are not easily separable, limiting their potential applications. In this work, by changing the preparation procedures, the calcium alginate film can be easily detached from substrates due to the weak nature of the forces between the calcium alginate film and the substrate. The weaker adhesion may be due to the fast consumption of CaCl_2_ in crosslinking with sodium alginate and the lack of anchors between film and substrate. As shown in Fig. 4a, the calcium alginate film was detached successfully from glass substrate and form free-standing flexible membrane with high flexibility. Besides planar free-standing membrane, some materials with different shapes can also be used as substrate, and after the detachment of films, various free-standing calcium alginate materials can be obtained. By using glass tube as template, a freestanding calcium alginate tube can be obtained (Fig. 4b).

Because the calcium alginate coatings impart self-cleaning property against oil contamination on various substrates, it may have many applications in condiment bottles. In this proof of concept, the oil red labeled salad oil was introduced into bottles as shown in Fig. 5. For the bottle with water-swollen calcium alginate film as inner coating, oil can slide smoothly inside bottle without a trace or stain left behind, while the uncoated bottle was completely stained by the colored oil. Since alginate is a safe food additive, the calcium alginate coatings will be valuable in surface modifications of food container.

In conclusion, we have developed a simple, environmental friendly and scalable route to fabricate oil-repellent hydrogel coatings. The as-fabricated coatings exhibited excellent underwater superoleophobicity and oil droplet can easy slide across and off the substrate, which endows self-cleaning property against oil contaminant. The proposed approach is versatile in fabricating oil-repellent coating into various material substrates, and not limited by their sizes, shapes and chemical properties. Moreover, this approach can be expanded to other material system. The oil-repellent coatings may have highly impact in daily life, such as self-cleaning interior bottle coatings or in food packaging to reduce food wastage.

## Experimental Section

### Fabrication of calcium alginate film

The substrates for film fabrication including steel foils, silicon wafers, glass slides, polyethylene terephthalate (PET) films, mica films were cleaned by distilled water, acetone, and ethanol successively. After treated by plasma, the substrates were coated by CaCl_2_ (0.010 g/mL) and sodium alginate (0.015 g/mL). By changing the coating sequence of sodium alginate and CaCl_2_, the free-standing calcium alginate film can be easily obtained by peeling off from the substrates.

### Instruments and Characterization

A field-emission scanning electron microscope (JSM-6700F, Japan) was used for characterizing the morphologies of the as-prepared film. Contact angles (CA) were measured on an OCA20 system (Data-Physics, Germany) at ambient temperature. The tested droplet (water, 1, 2-dichloroethane or other tested oils, 2 μL) was syringed out and dropped carefully onto the surfaces. The underwater oil contact angles were measured by immersing the surfaces in distilled water. The average CA values were obtained by measuring at five different positions on the same sample. The sliding angles were determined by slowly increasing the sliding angle of sliding stage until a given droplet begins to slide along the surface.

### Materials

Oil red O were purchased from Sigma-Aldrich. Silicon oil with different viscosities (5 cst) was purchased from DowCorning. Salad oil (JinLongYu) was purchased from supermarket. All other reagents including sodium alginate, CaCl_2_ and solvents were of analytical reagent grade and obtained from Sinopharm Chemical Reagent co. Ltd, China. They were used without further purification. All solutions were prepared with ultrapure water (Milli-Q, 18.2 MΩcm).

## Additional Information

**How to cite this article**: Xu, L.-P. *et al*. A Green Route for Substrate-Independent Oil-Repellent Coatings. *Sci. Rep.* **6**, 38016; doi: 10.1038/srep38016 (2016).

**Publisher's note:** Springer Nature remains neutral with regard to jurisdictional claims in published maps and institutional affiliations.

## Supplementary Material

Supplementary Information

## Figures and Tables

**Figure 1:**
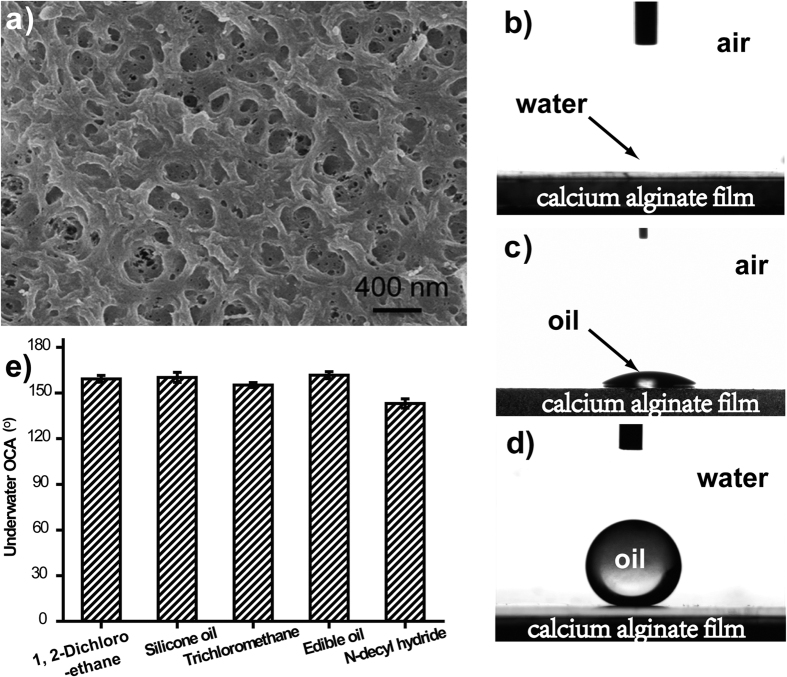
(**a**) SEM image of calcium alginate film; (**b**) A photograph of a water droplet (2 μ L) on calcium alginate film in air with a contact angle of almost 0°; (**c**) A photograph of an oil droplet (1, 2-Dichloroethane, DCL, c.a. 2 μ L) on the calcium alginate film in air with an oil contact angle of 33.7 ± 6.2°; (**d**) A photograph of an oil droplet (DCL, c.a. 2 μ L) on the calcium alginate film under water with an oil contact angle of 159.3 ± 2.2°; (**e**) Underwater superoleophobicity of calcium alginate film in the oil/water/solid three-phase system for various oils in terms of their contact angles.

**Figure 2:**
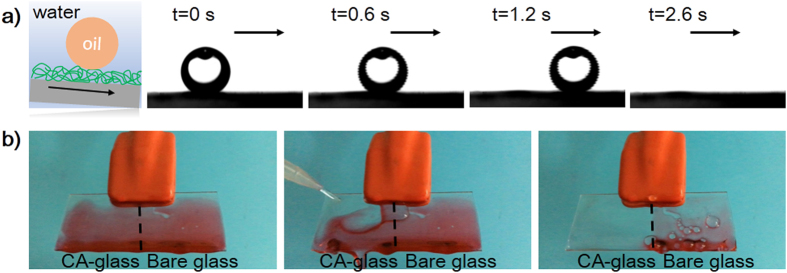
(**a**) A DCL oil droplet (2 μL) can easily slide off a calcium alginate film underwater with sliding angle of less than 2°; (**b**) The self-cleaning property of an a calcium alginate film after absorbing water for 10 min, silicone oil dyed with oil red was used as the dirt.

**Figure 3:**
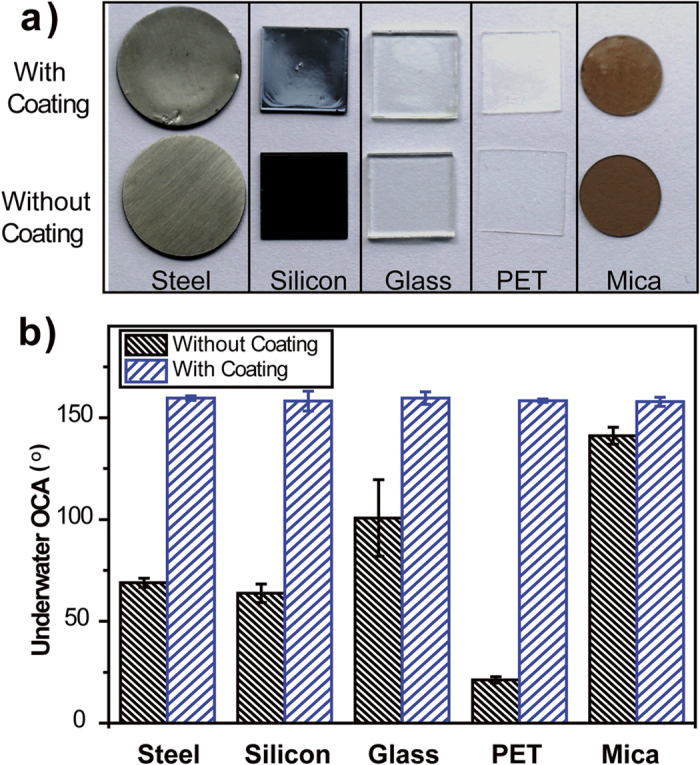
(**a**) Photograph of various planar substrates without (upper) and with (lower) calcium alginate coatings; (**b**) underwater OCA values of several substrates without and with calcium alginate coatings.

**Figure 4:**
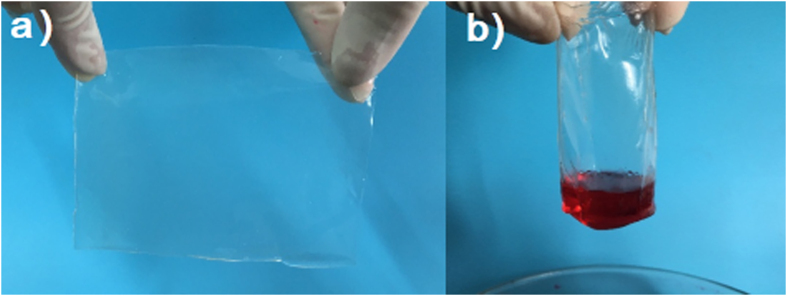
Photographs of substrate-independent calcium alginate materials with various shapes. (**a**) Freestanding planar calcium alginate film and (**b**) freestanding calcium alginate tube.

**Figure 5:**
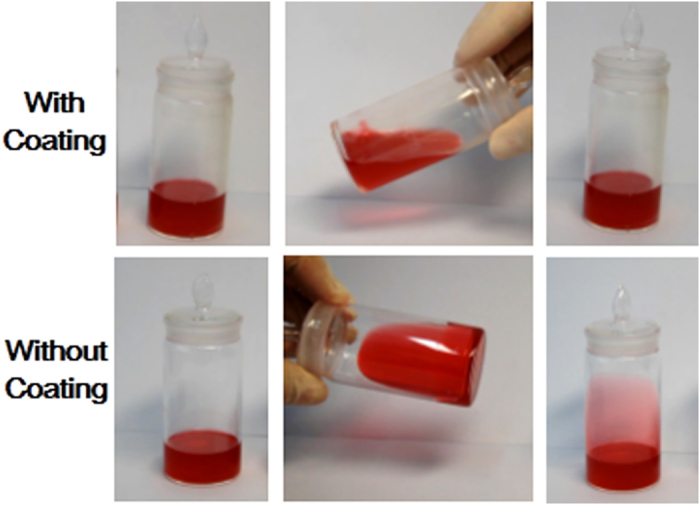
Images of salad oil (dyed with oil red) in (**a**) a bottle with calcium alginate coating and (**b**) a traditional bottle.

## References

[b1] Cao L., Price T. P., Weiss M. & Gao D. Super water- and oil-repellent surfaces on intrinsically hydrophilic and oleophilic porous silicon films. Langmuir 24, 1640–1643 (2008). 18198916 10.1021/la703401f

[b2] Leng B., Shao Z., de With G. & Ming W. Superoleophobic Cotton Textiles. Langmuir 25, 2456–2460 (2009). 19199744 10.1021/la8031144

[b3] Tuteja A., Choi W., Mabry J. M., McKinley G. H. & Cohen R. E. Robust omniphobic surfaces. Proc Natl Acad Sci 105, 18200–18205 (2008). 19001270 10.1073/pnas.0804872105 PMC2587612

[b4] Deng X., Mammen L., Butt H.-J. & Vollmer D. Candle Soot as a Template for a Transparent Robust Superamphiphobic Coating. Science 335, 67–70 (2012). 22144464 10.1126/science.1207115

[b5] Tuteja A. . Designing Superoleophobic Surfaces. Science 318, 1618–1622 (2007). 18063796 10.1126/science.1148326

[b6] Liu X. . Clam’s Shell Inspired High-Energy Inorganic Coatings with Underwater Low Adhesive Superoleophobicity. Adv Mater 24, 3401–3405 (2012). 22648962 10.1002/adma.201200797

[b7] Cheng Q. . An underwater pH-responsive superoleophobic surface with reversibly switchable oil-adhesion. Soft Matter 8, 2740–2743 (2012).

[b8] Liu H., Zhang X., Wang S. & Jiang L. Underwater Thermoresponsive Surface with Switchable Oil-Wettability between Superoleophobicity and Superoleophilicity. Small 11, 3338–3342 (2015). 25689605 10.1002/smll.201403190

[b9] Wong T. S. . J. Bioinspired self-repairing slippery surfaces with pressure-stable omniphobicity. Nature 477, 443–447 (2011). 21938066 10.1038/nature10447

[b10] Joly L. & Biben T. Wetting and friction on superoleophobic surfaces. Soft Matter, 2549–2557 (2009).

[b11] Wang S. T., Liu K. S., Yao X. & Jiang L. Bioinspired Surfaces with Superwettability: New Insight on Theory, Design, and Applications. Chem Rev 115, 8230–8293 (2015). 26244444 10.1021/cr400083y

[b12] Wang Z., Hou D. & Lin S. Composite Membrane with Underwater-Oleophobic Surface for Anti-Oil-Fouling Membrane Distillation. Environ Sci Technol 50, 3866–3874 (2016). 26958985 10.1021/acs.est.5b05976

[b13] Matsubayashi T. . A facile method of synthesizing size-controlled hollow cyanoacrylate nanoparticles for transparent superhydrophobic/oleophobic surfaces. Rsc Advances 6, 15877–15883 (2016).

[b14] Lee J., Boo C., Ryu W.-H., Taylor A. D. & Elimelech M. Development of Omniphobic Desalination Membranes Using a Charged Electrospun Nanofiber Scaffold. ACS Appl Mater Interfaces 8, 11154–11161 (2016). 27065300 10.1021/acsami.6b02419

[b15] Chen P.-C. & Xu Z.-K. Mineral-Coated Polymer Membranes with Superhydrophilicity and Underwater Superoleophobicity for Effective Oil/Water Separation. Sci Rep 3, 2776 (2013). 24072204 10.1038/srep02776 PMC3784956

[b16] Zhang L., Zhong Y., Cha D. & Wang P. A self-cleaning underwater superoleophobic mesh for oil-water separation. Sci Rep 3, 2326 (2013). 23900109 10.1038/srep02326 PMC3728594

[b17] Sun Y., Chen M., Zhou S., Hu J. & Wu L. Controllable Synthesis and Surface Wettability of Flower-Shaped Silver Nanocube-Organosilica Hybrid Colloidal Nanoparticles. ACS Nano 9, 12513–12520 (2015). 26564332 10.1021/acsnano.5b06051

[b18] Chen K., Zhou S., Yang S. & Wu L. Fabrication of All-Water-Based Self-Repairing Superhydrophobic Coatings Based on UV-Responsive Microcapsules. Adv Funct Mater 25, 1035–1041 (2015).

[b19] Kota A. K., Li Y., Mabry J. M. & Tuteja A. Hierarchically structured superoleophobic surfaces with ultralow contact angle hysteresis. Adv Mater 24, 5838–5843 (2012). 22930526 10.1002/adma.201202554

[b20] Tuteja A., Choi W., McKinley G. H., Cohen R. E. & Rubner M. F. Design parameters for superhydrophobicity and superoleophobicity. MRS Bull 33, 752–758 (2008).

[b21] Kota A. K., Kwon G., Choi W., Mabry J. M. & Tuteja A. Hygro-responsive membranes for effective oil–water separation. Nat Commun 3, 1025 (2012). 22929782 10.1038/ncomms2027

[b22] Kim P. . Liquid-infused nanostructured surfaces with extreme anti-ice and anti-frost performance. ACS Nano 6, 6569–6577 (2012). 22680067 10.1021/nn302310q

[b23] Sunny S., Vogel N., Howell C., Vu T. L. & Aizenberg J. Lubricant-Infused Nanoparticulate Coatings Assembled by Layer-by-Layer Deposition. Adv Funct Mater 24, 6658–6667 (2014).

[b24] Yao X. . Adaptive fluid-infused porous films with tunable transparency and wettability. Nat Mater 12, 529–534 (2013). 23563739 10.1038/nmat3598

[b25] Xiao L. . Slippery Liquid-Infused Porous Surfaces Showing Marine Antibiofouling Properties. ACS Appl Mater Interfaces 5, 10074–10080 (2013). 24067279 10.1021/am402635p

[b26] Howell C. . Self-replenishing vascularized fouling-release surfaces. ACS Appl Mater Interfaces 6, 13299–13307 (2014). 25006681 10.1021/am503150y

[b27] Jin H., Tian X., Ikkala O. & Ras R. H. A. Preservation of Superhydrophobic and Superoleophobic Properties upon Wear Damage. ACS Appl Mater Interfaces 5, 485–488 (2013). 23339565 10.1021/am302541f

[b28] Xu L.-P. . Nacre-Inspired Design of Mechanical Stable Coating with Underwater Superoleophobicity. ACS Nano 7, 5077–5083 (2013). 23701041 10.1021/nn400650f

[b29] Liu M., Wang S. T., Wei Z., Song Y. & Jiang L. Bioinspired Design of a Superoleophobic and Low Adhesive Water/Solid Interface. Adv Mater 21, 665–669 (2009).

[b30] Xu L.-P. . An Ion-Induced Low-Oil-Adhesion Organic/Inorganic Hybrid Film for Stable Superoleophobicity in Seawater. Adv Mater 25, 606–611 (2013). 23132773 10.1002/adma.201203461

[b31] Cheng Z. . Underwater superoleophilic to superoleophobic wetting control on the nanostructured copper substrates. ACS Appl Mater Interfaces 5, 11363–11370 (2013). 24083992 10.1021/am403595z

[b32] Chen K., Zhou S. & Wu L. Self-Healing Underwater Superoleophobic and Antibiofouling Coatings Based on the Assembly of Hierarchical Microgel Spheres. ACS Nano 10, 1386–1394 (2016). 26687925 10.1021/acsnano.5b06816

[b33] Manna U. & Lynn D. M. Synthetic Surfaces with Robust and Tunable Underwater Superoleophobicity. Adv Funct Mater 25, 1672–1681 (2015).

[b34] Rowley J. A., Madlambayan G. & Mooney D. J. Alginate hydrogels as synthetic extracellular matrix materials. Biomaterials 20, 45–53 (1999). 9916770 10.1016/s0142-9612(98)00107-0

[b35] Lee K. Y. & Mooney D. J. Alginate: Properties and biomedical applications. Prog Polym Sci 37, 106–126 (2012). 22125349 10.1016/j.progpolymsci.2011.06.003 PMC3223967

[b36] Li Z. S., Ramay H. R., Hauch K. D., Xiao D. M. & Zhang M. Q. Chitosan-alginate hybrid scaffolds for bone tissue engineering. Biomaterials 26, 3919–3928 (2005). 15626439 10.1016/j.biomaterials.2004.09.062

[b37] Saha S., Pal A., Kundu S., Basu S. & Pal T. Photochemical Green Synthesis of Calcium-Alginate-Stabilized Ag and Au Nanoparticles and Their Catalytic Application to 4-Nitrophenol Reduction. Langmuir 26, 2885–2893 (2010). 19957940 10.1021/la902950x

[b38] Sharma S., Sanpui P., Chattopadhyay A. & Ghosh S. S. Fabrication of antibacterial silver nanoparticle-sodium alginate-chitosan composite films. RSC Advances 2, 5837–5843 (2012).

[b39] Neto A. I. . Fabrication of Hydrogel Particles of Defined Shapes Using Superhydrophobic-Hydrophilic Micropatterns. Adv Mater, 10.1002/adma.201602350 (2016). 27332997

[b40] Shen L. . Asymmetric free-standing film with multifunctional anti-bacterial and self-cleaning properties. ACS Appl Mater Interfaces 4, 4476–4483 (2012). 22947922 10.1021/am301118f PMC4111538

[b41] Zhao X., Su Y., Liu Y., Lip Y. & Jiang Z. Free-Standing Graphene Oxide-Palygorskite Nanohybrid Membrane for Oil/Water Separation. ACS Appl Mater Interfaces 8, 8247–8256 (2016). 26978041 10.1021/acsami.5b12876

