# Supplementary material for: A Green Route for Substrate-Independent Oil-Repellent Coatings
Source: Sci Rep. 2016 Nov 29;6:38016. doi: 10.1038/srep38016 (PMC5126561; doi:10.1038/srep38016)
Supplement: Supplementary Information [file srep38016-s1.doc]

A Green Route for Substrate-Independent Oil-Repellent Coatings

Li-Ping Xu,^† ,*^ Da Han, ^†^ Xiuwen Wu, ^†^ Qingqing Zhang, ^†^ Xueji Zhang, ^†^ Shutao Wang^‡,*^

†Research Center for Bioengineering and Sensing Technology, School of Chemistry and Biological Engineering, University of Science & Technology Beijing, Beijing 100083, P.R. China.

‡ Laboratory of Bioinspired Smart Interface Science, CAS Center for Excellence in Nanoscience, Technical Institute of Physics and Chemistry, Chinese Academy of Sciences, Beijing 100190, P.R. China.

*Correspondence to [stwang@mail.ipc.ac.cn](mailto:stwang@mail.ipc.ac.cn) or xuliping@ustb.edu.cn.


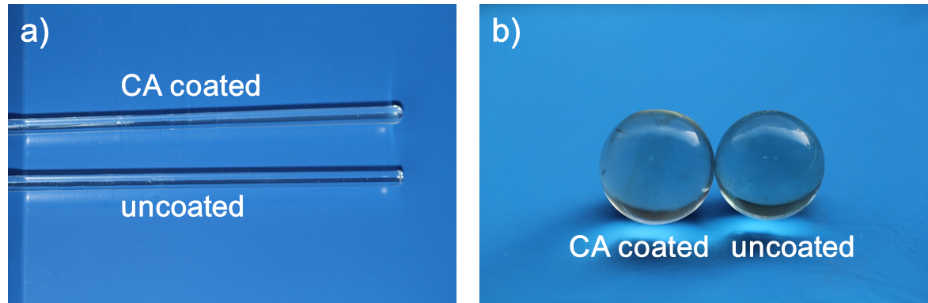


Fig. S1 Photographs of a) glass rods and b) glass beads with and without calcium alginate coating.
